# A cuckoo COVID coincidence?

**DOI:** 10.1183/13993003.03236-2020

**Published:** 2020-10-15

**Authors:** James D. Chalmers, Mike Lonergan

**Affiliations:** Division of Molecular and Clinical Medicine, School of Medicine, University of Dundee, Dundee, UK

## Abstract

We are grateful to A.H. Morice for his reminder of the limits of observational data, and the need for caution in its interpretation. And we accept that it is unlikely to ever be proved conclusively that the dramatic restrictions imposed as countries locked down caused the dramatic changes in epidemic trajectories, even though the epidemiological changes seemed to follow the behavioural changes. However, there is also a large logical leap from observing the Swedish data to inferring that social distancing “measures can only have had a minor effect”.

## Abstract 2

**There are limitations to inferring causality from observational data, but lockdowns have almost certainly saved lives worldwide** https://bit.ly/2YWAhhl

From the authors:

We are grateful to A.H. Morice for his reminder of the limits of observational data, and the need for caution in its interpretation. And we accept that it is unlikely to ever be proved conclusively that the dramatic restrictions imposed as countries locked down caused the dramatic changes in epidemic trajectories, even though the epidemiological changes seemed to follow the behavioural changes. However, there is also a large logical leap from observing the Swedish data to inferring that social distancing “measures can only have had a minor effect”.

The decisions of the Swedish government certainly differed from those of other countries. The Swedish decline in daily mortalities was also slower than in the rest of Europe, including the UK. It is true that that those extra deaths cannot be conclusively proved to be the responsibility of the public health policy of the Swedish government. However, Sweden is not isolated. The natural comparators for the Swedish experience are its neighbours: Denmark, Norway and Finland. All of them experienced far lower, and more rapidly declining, mortality rates. Many Swedish people modified their behaviour substantially, following and even exceeding governmental recommendations that resembled many of the restrictions imposed in other countries [1].

The USA is another western country that did not follow the pattern of locking down tightly. And its patchwork of decisions and timings may be the nearest data we will have to trials of the effects of different measures. But even they cannot prove causation.

So there remain at least four possibilities: the lockdowns changed the course of the epidemic; the epidemic was halted by behavioural changes that would have occurred without the governmental lockdown (effectively a people's lockdown); spring reduced transmission sufficiently to stop the virus around the time of each European country's lockdown; or, in each country, changes occurred in the virus around the time of lockdown. Until next winter, choosing between them will largely be a matter of belief, though it is unclear how an argument for the centrality of seasonality would explain the apparent successes of autumnal control measures in Australia and New Zealand.

One response to this uncertainty would be to give up on statistical analysis and modelling, and fall back on common sense to deal with this unprecedented situation. Some instincts, such as those to avoid crowds of strangers and individuals who show signs of sickness, are certainly appropriate for personal safety within a pandemic, but it is not obvious how they would inform decisions on purchasing ventilators or creating extra hospital capacity. And, while the Nightingale Hospitals may feel like wasted money now, they were an insurance policy. Regretting the purchase of insurance because we didn't need to make a claim rather misses its point.

While we agree with A.H. Morice about the importance of assumptions, we do believe models can extract useful information from, even limited, data. We failed if our models [2] were “indecipherable”. They were very simple in concept: as directly transmissible diseases spread exponentially while they are rare and other conditions are constant. The proportion of the population that caught coronavirus disease 2019 (COVID-19) was relatively small, and the main methodological novelty in our paper was estimating exponential rates of decline after each country's peak as well as exponential growth rates before it. Separate models were fitted for each country, partly because, as A.H. Morice says, their “surety of data collection varies”.

And we also agree that all, certainly including our, model results need to be treated with caution: they are simplified representations rather than reality. It wasn't hubris that led us to say our “estimates are incompatible with a return to previous activities post ‘lockdown’”, but a recognition that they were estimates rather than definitive truth.

There is one issue that we clearly missed: while the change from rapid increases to slow declines does suggest that the behavioural changes were only just sufficient, as we stated, we did not acknowledge that some of the restrictions might have absolutely no effect. So, if banning sunbathing in parks, or restricting people to one 1-h period of exercise per day, made no difference to disease transmission they were irrelevant. We should have clearly stated that it was a majority out of those behavioural changes that affected disease transmission that required continuation. Working out which these were effective will be difficult, especially as people are responding in very different ways to the easing of restrictions. Without data, statistical analysis, and, probably, modelling we are unlikely to be able to untangle those effects. And the argument as to whether it is better to do too much or too little about a public health crisis is unanswerable in the abstract: gradually, as information becomes available and is analysed, we can hope to tune the distribution of resources between COVID-19 and other priorities. But, whatever happens, we will remain grateful to A.H. Morice for his contribution to keeping us honest.

## Shareable PDF

10.1183/13993003.03236-2020.Shareable1 This one-page PDF can be shared freely online.
Shareable PDF ERJ-03236-2020.Shareable

## Supplementary Material

ERJ-03236-2020.Shareable.pdf
