# Supplementary material for: A cuckoo COVID coincidence?
Source: Eur Respir J. 2020 Oct 15;56(4):2003236. doi: 10.1183/13993003.03236-2020 (PMC7487271; doi:10.1183/13993003.03236-2020)
Supplement: Supplementary file 1 [file ERJ-03236-2020.Shareable.pdf]

SHAREABLE PDF

A cuckoo COVID coincidence?

James D. Chalmers and Mike Lonergan

Affiliation: Division of Molecular and Clinical Medicine, School of Medicine, University of Dundee, Dundee, UK.

Correspondence: Mike Lonergan, Division of Molecular and Clinical Medicine, University of Dundee, Ninewells Hospital and Medical School, Dundee, DD1 9SY, UK. E-mail: m.lonergan@dundee.ac.uk

@ERSpublications
There are limitations to inferring causality from observational data, but lockdowns have almost certainly saved lives worldwide https://bit.ly/2YWAhhl

Cite this article as: Chalmers JD, Lonergan M. A cuckoo COVID coincidence?. *Eur Respir J* 2020; 56: 2003236 [https://doi.org/10.1183/13993003.03236-2020].

This single-page version can be shared freely online.

From the authors:

We are grateful to A.H. Morice for his reminder of the limits of observational data, and the need for caution in its interpretation. And we accept that it is unlikely to ever be proved conclusively that the dramatic restrictions imposed as countries locked down caused the dramatic changes in epidemic trajectories, even though the epidemiological changes seemed to follow the behavioural changes. However, there is also a large logical leap from observing the Swedish data to inferring that social distancing “measures can only have had a minor effect”.

Copyright ©ERS 2020.. This version is distributed under the terms of the Creative Commons Attribution Non-Commercial Licence 4.0.
